# PReS-FINAL-2360: Severe disease course of pediatric-onset granulomatosis with polyangiitis as compared to adult patients: a long-term, single center, follow up study

**DOI:** 10.1186/1546-0096-11-S2-P350

**Published:** 2013-12-05

**Authors:** G Minardo, V Carraro, G Martini, L Murer, F Vittadello, F Schiavon, F Zulian

**Affiliations:** 1 Department of Pediatrics, University of Padua, Padua, Italy; 2 Division of Rheumatology, University of Padua, Padua, Italy

## Introduction

Granulomatosis with polyangiitis (GPA) is a rare disease in childhood. Treatment strategies and clinical approach are still mostly derived from adult GPA studies.

## Objectives

The present study was aimed to compare disease onset and course, therapeutic approach and clinical outcome of two cohorts of children and adults affected by GPA.

## Methods

The study included children and adult patients selected on the basis of complete data set and follow up of at least one year, followed at the pediatric and adult Rheumatology Centers, University Hospital of Padua, over a period of 20 years (1993-2013). GPA was diagnosed according to the ACR and EULAR/PRES criteria. Clinical features, instrumental findings, laboratory parameters and therapeutic regimens of both cohorts were analyzed at diagnosis, six months later (T6) and at the last follow-up visit. Disease activity was assessed by the Birmingham Vasculitis Activity Score (BVAS) modified for GPA.

## Results

Ten children with mean age at disease onset of 10.3 years (range 3-15) entered the study, 6/9 were female. Mean follow-up time was 8.4 years (range 2.5-18). Of the 23 adults 65.3% were female, mean age at onset 53 years (range 37-71) and mean follow-up 1.9 years (range 1-3.5). At disease onset, BVAS, clinical features and laboratory parameters of the two cohorts were not significantly different. BVAS decreased more slowly in children (p 0.002). As for internal organs involvement, renal disease was significantly higher at T6 and persisted over time in children (p 0.003). Similarly, pulmonary disease remained elevated at T6 while decreased over 50% in adults (p 0.01). At the last F/U visit, eye involvement was present in 44% of children while no adult showed signs of ocular disease (p 0.004). Regarding to the treatment strategies, immunosuppressive drugs were more widely used in children at diagnosis (p 0.06) and biological agents were used at an earlier disease stage than adults. Despite the longer follow up, all children were still on treatment at the last visit while 17% of adults were off therapy.

## Conclusion

Adults and children with GPA had similar disease activity at onset. However, childhood GPA had a more severe-course due to persistent renal, pulmonary and eye involvement. Lower disease activity was obtained with a more aggressive treatment approach although no pediatric patients reached a treatment-free remission.

## Disclosure of interest

None declared.

